# Yangtze River, an insignificant genetic boundary in tufted deer (*Elaphodus cephalophus*): the evidence from a first population genetics study

**DOI:** 10.7717/peerj.2654

**Published:** 2016-11-08

**Authors:** Zhonglou Sun, Tao Pan, Hui Wang, Mujia Pang, Baowei Zhang

**Affiliations:** 1 School of Life Sciences, Anhui University, Hefei, Anhui, China; 2 School of Biosciences, Cardiff University, Cardiff, United Kingdom

**Keywords:** *Elaphodus cephalophus*, Yangtze River, Geographical barrier, Genetic structure, Gene flow, Population decline

## Abstract

Great rivers were generally looked at as the geographical barrier to gene flow for many taxonomic groups. The Yangtze River is the third largest river in the world, and flows across South China and into the East China Sea. Up until now, few studies have been carried out to evaluate its effect as a geographical barrier. In this study, we attempted to determine the barrier effect of the Yangtze River on the tufted deer (*Elaphodus cephalophus*) using the molecular ecology approach. Using mitochondrial DNA control region (CR) sequences and 13 nuclear microsatellite loci, we explored the genetic structure and gene flow in two adjacent tufted deer populations (Dabashan and Wulingshan populations), which are separated by the Yangtze River. Results indicated that there are high genetic diversity levels in the two populations, but no distinguishable haplotype group or potential genetic cluster was detected which corresponded to specific geographical population. At the same time, high gene flow was observed between Wulingshan and Dabashan populations. The tufted deer populations experienced population decrease from 0.3 to 0.09 Ma BP, then followed by a distinct population increase. A strong signal of recent population decline (*T* = 4,396 years) was detected in the Wulingshan population by a Markov-Switching Vector Autoregressions(MSVAR) process population demography analysis. The results indicated that the Yangtze River may not act as an effective barrier to gene flow in the tufted deer. Finally, we surmised that the population demography of the tufted deer was likely affected by Pleistocene climate fluctuations and ancient human activities.

## Introduction

Natural landscape features, such as rivers, can function as genetic boundaries and shape the population structure of animals because they can act as an important geographical barrier to dispersal and gene flow (Funk et al., 2001; Whiteley, Spruell & Allendorf, 2004; Coulon et al., 2004; Hartl et al., 2005; Coulon et al., 2006; Wang et al., 2015). In addition, rivers have been found to impact on genetic structure and population divergence in various species (Gaines et al., 1997; Nupp & Swihart, 1998; Grubb, 2001; Bergl & Vigilant, 2007). Up to now, rivers have been identified as a barrier to gene flow in several taxonomic groups, such as small mammals (Lugon-Moulin & Hausser, 2002), reptiles (Mockford et al., 2007; Zhao et al., 2011), and even in birds (Fernandes et al., 2013). In Europe, large or small rivers obstruct the dispersal and movement in the European badger (*Meles meles*) (Frantz et al., 2010). Even for some large mammals, such as the grey wolf, *Canis lupus* (Carmichael et al., 2001), the giant panda, *Ailuropoda melanoleuca* (Zhu et al., 2011), and the white-tailed deer, *Odocoileus virginianus* (Robinson et al., 2012), rivers still presented substantial limits to dispersal and gene flow in spite of their high mobility. And what is more, Hayes & Sewlal (2004) found the Amazon River to be an effective dispersal barrier for the antbirds (Thamnophilidae). Generally, rivers may actually facilitate gene flow in some amphibians (Spear et al., 2005).

The Yangtze River, the third largest river in the world, flows from west to east before entering the East China Sea (Chen et al., 2001). The wide channel, turbulent flow and many steep cliffs caused it to become a natural geographical barrier for some animals, including the house mice, *Mus musculus* (Jing et al., 2014), and early humans (Lynn, 1997; Chu et al., 1998; Su et al., 1999). Even some tributaries of the Yangtze River also function as important geographical barrier. For example, the Dadu River had acted as the significant genetic boundary between the Daxiangling population and Xiaoxiangling population of the giant panda (Zhu et al., 2011). In Southwestern China, the lower reach of the Yalong River had a significant barrier effect on the plateau wood frog, *Rana kukunoris* (Zhao, Dai & Fu, 2009). However, for some large animals, the Yangtze River was not an insurmountable geographical barrier. For example, the weak population differentiations between South China population and North China population of the wild boar (*Sus scrofa*) indicated that the Yangtze River did not constitute an effective geographic barrier to the wild boar (Zhang et al., 2008). Similarly, Shi et al. (2010) revealed a high gene flow level between the two adjacent Chinese muntjac (*Muntiacus reevesi*) populations separated by Yangtze River, which indicated that Yangtze River was not an effective geographical barrier for the Chinese muntjac neither.

**Figure 1:**
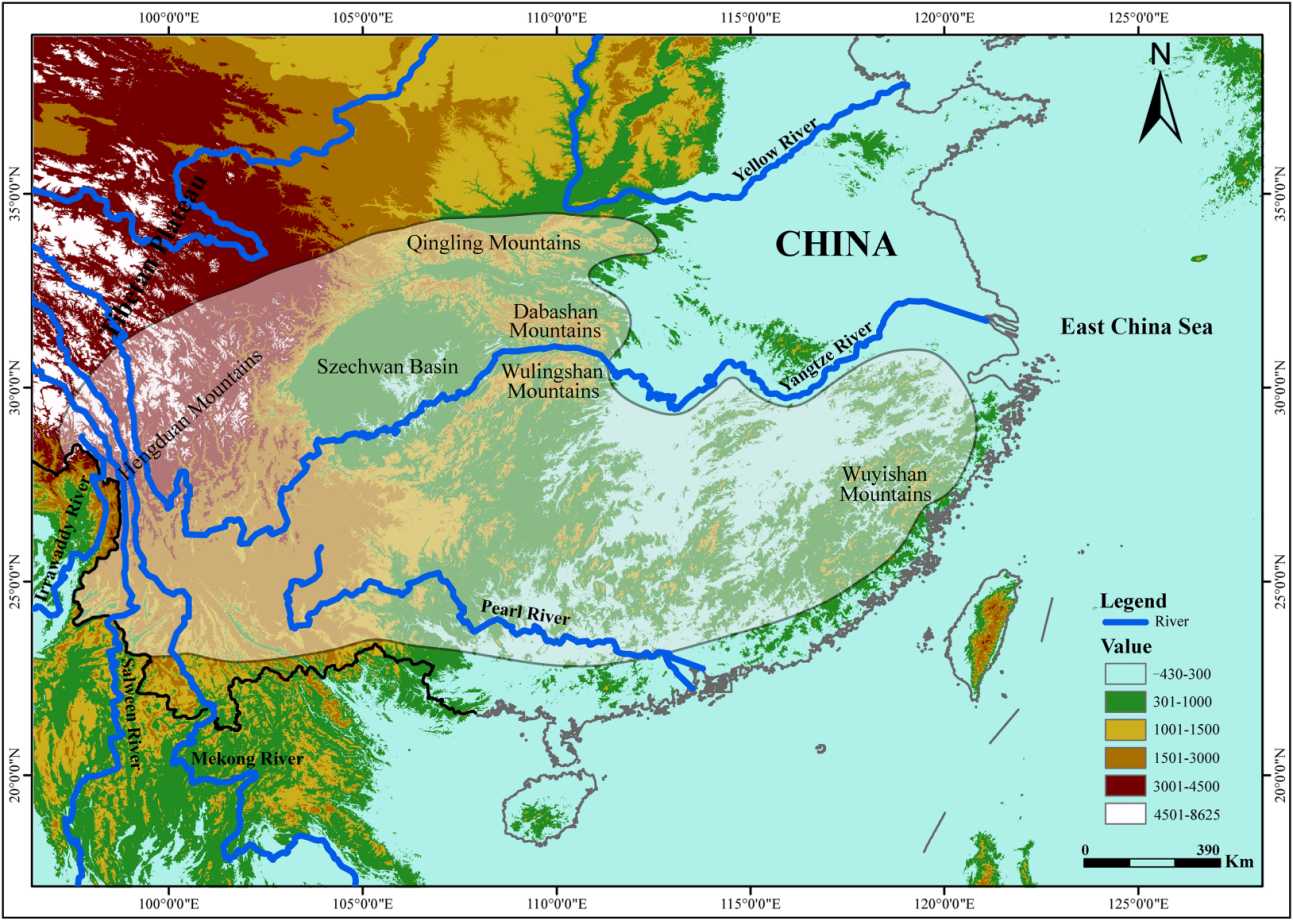
The geographic distribution of the tufted deer (*Elaphodus cephalophus*). The highlighted area with shading represent the tufted deer’s distribution region (Sheng et al., 1992).

The tufted deer (*Elaphodus cephalophus*) is a native species to central and southwest China (Fig. 1), from the Hengduan Mountains, the peaks around the Szechwan Basin and the Qingling Mountains southeastward to the Wuyishan Mountains (Sheng & Lu, 1982; Sheng et al., 1992; Wang, 2003). The tufted deer is a timid animal, mainly solitary or found in pairs. It inhabits mountainous terrain with good cover (Sheng et al., 1992). In recent decades, the wild populations of tufted deer have declined sharply because of overhunting by locals for meat and leather and habitat degradation (Zhang & Wei, 2007). Currently, tufted deer is categorized as “Near Threatened” (NT) by International Union for Conservation of Nature (2015). Understanding the evolutionary history and population demography along with their current genetic structure and diversity, including geographic variations, enables effective conservation and management of endangered species (Avise, 1989; Smith, Bruford & Wayne, 1993; O’Brien, 1994; Beaumont & Bruford, 1999; Osentoski et al., 2002; Zhang et al., 2007; Bu, Liu & Nie, 2014). Unfortunately, effective conservation measures have not yet to be implemented for the tufted deer (Wu et al., 2007) and, up to now, few studies explored the genetic profile of the tufted deer and the possible effects that the Yangtze River imposed on it.

It is well known that patterns of dispersal or gene flow can greatly affect the evolutionary and adaptive potential of populations (Slatkin, 1987). In the present study, we attempt to explore the barrier effect of the Yangtze River on the tufted deer. It was recorded that Wulingshan and Dabashan mountains harbor large populations of tufted deer (Sheng et al., 1992), that are isolated from each other by the Yangtze River (Fig. 1), so it should be the optimal area to carry out this study. In this study, we integrated data derived from the mtDNA control region (CR) sequence and 13 nuclear microsatellite loci, and investigated the genetic profile of the tufted deer populations. Furthermore, we evaluated the effect that the Yangtze River had on the tufted deer’s genetic structure and gene flow. The findings should be useful for conservation and management strategies and prioritize the management outcomes in other populations.

## Materials and Methods

### Ethics statement

In the present study, samples from carcass of naturally deceased animals were collected within the realm of an ongoing tufted deer research project. Our experimental procedures were specifically approved by the Animal Research Ethics Committee of Anhui University (Animal Ethics number: AHU3110).

### Sample collection and DNA extraction

A total of 59 dried skin samples were collected, including 41 from Wulingshan Mountains (WLS, located in the south of the Yangtze River) and 18 from Dabashan Mountains (DBS, located in the north of the Yangtze River) (Fig. 1).

Total genomic DNA was extracted from dried skin using the standard phenol-chloroform protocol, as described by Sambrook & Russell (2001).

### PCR amplification, sequencing and genotyping

Based on the complete mtDNA sequence of the tufted deer (GenBank accession no. DQ873526) (Pang et al., 2008), we designed a pair of specific primers. Forward primer L15340 (5′-GTATACTCAATACACTGGTCTTGT-3′) located in tRNA^Pro^ gene, and reverse primer H167 (5′-GTGCTTGATACCAGCTCCTCT-3′) located in 12S rRNA gene. DNA fragments measuring approximately 1,110 bp, including the complete mtDNA CR (about 915 bp) along with about 185 bp of its flanking sequences, were amplified for all samples.

The mtDNA CR was amplified in a 50 μL reaction mixture: 100–200 ng of genomic DNA, 25 μL 2 × Easy *Taq* Polymerase Chain Reaction (PCR) SuperMix polymerase (TransGen Biotech, containing 1.25U Ex *Taq*, 0.4 mM dNTP, 4 mM Mg^2+^) and 0.4 μM of primers. Thermal cycling consisted of a denaturation step at 94 °C for 5 min, followed by 30 cycles of denaturation (94 °C, 30 s), annealing (52 °C, 30 s) and extension (72 °C, 60 s) and a final extension step of 10 min at 72 °C. PCR products were gel-purified in 1% agarose, excised from the gel, and purified with a QIAquick Gel Extraction Kit (Qiagen). Finally, PCR products were sequenced on ABI PRISM 3730 DNA sequencer.

In the present study, 13 high polymorphic loci (Mreg03, Mreg22, Mreg25, Mreg26, Mre35, Mre39, Mre49, Mre61, Mreg143, Mreg252, Mreg260, Mreg283, Mreg284) developed for the tufted deer (Wang et al., 2013; Wang et al., 2014) were utilized. Microsatellite PCR was performed in a 20 μL reaction comprising 100–200 ng of genomic DNA, 10 μL 2 × Easy *Taq* PCR Supermix (TransGen Biotech, containing 1.25U Ex *Taq*, 0.4 mM dNTP, 4 mM Mg^2+^) and 0.2 μM of each primer (forward primer fluorescently labeled with Carboxyfluorescein (FAM), Hexachlorofluorescein (HEX) or Tetramethylrhodamine (TAMRA)). Thermal cycling consisted of a denaturation step at 94 °C for 5 min, followed by 30 cycles of denaturation (94 °C, 30 s), annealing (52 °C, 30 s) and extension (72 °C, 40 s) and a final extension step of 10 min at 72 °C. PCR products were separated on an ABI PRISM 3730 genetic analyzer (Applied Biosystems) with a GS500 size standard, and analyzed using GENEMARKER (version 1.3, SoftGenetics LLC).

### Data analysis

DNA fragments were aligned using the software Clustal X (Thompson et al., 1997) and examined visually. Nucleotide diversity and haplotype diversity were calculated according to Nei (1987), using the software DnaSP version 5 (Librado & Rozas, 2009), and the haplotypes sequence were deposited in GenBank (Accession no. KT152891–KT152929). In addition, we used NETWORK 4.5.0.2 (Polzin & Daneshmand, 2003) to draw a median-joining network to analyze the relationships among the detected haplotypes. Two neutrality tests, Tajima’s *D* test and Fu’s *Fs* test, were performed in ARLEQUIN 3.0 (Excoffier, Laval & Schneider, 2005). Pairwise *F*_ST_ value between populations were calculated by ARLEQUIN 3.0 (Excoffier, Laval & Schneider, 2005). Bayesian skyline plot (BSP) analysis of 52 tufted deer was conducted using BEAST1.6.2 (Drummond et al., 2005; Drummond & Rambaut, 2007). The BEAST analyses were conducted selecting HKY + I + G as the best-fit substitution by MrModelTest2 (Nylander, 2004). Two independent runs of Markov Chain Monte Carlo (MCMC) analyses for 10,000,000 generations were conducted with sampling every 1,000 generations and 10% of the initial samples were discarded as burn-in. In view of the mutation rate of mtDNA CR ranging from 1.7 to 2.5% per million years among different deer species (Hundertmark et al., 2002), we used the median value of 2% in the BSP analysis. Changes in female effective population size over time was analyzed in TRACER 1.5 and summarized as BSP (Drummond & Rambaut, 2007).

Micro-Checker v2.2.3 was used to detect the presence of null alleles and genotyping errors in microsatellite genotyping (Van Oosterhout et al., 2004). Linkage disequilibrium was tested with GENEPOP 4.2.1 (Rousset, 2008). The number of alleles (*N*_A_), observed heterozygosity (*H*_O_), expected heterozygosity (*H*_E_) and polymorphism information content (*PIC*) values for WLS, DBS and whole were calculated using GENETIX version 4.02 (Belkhir, Borsa & Chikhi, 2001). Allelic richness, an estimate of allelic diversity that compensates for unequal sample size, was calculated using FSTAT (Goudet, 2002) and averaged across loci. Pairwise *F*_ST_ value between populations were calculated by ARLEQUIN based on microsatellite data (Excoffier, Laval & Schneider, 2005). And total of 10,000 permutations were also performed to test the significance of pairwise population comparison in ARLEQUIN. Population structure was investigated with Bayesian cluster analysis using STRUCTURE 2.3.4 (Pritchard, Stephens & Donnelly, 2000; Falush, Stephens & Pritchard, 2003). Ten independent runs of K = 1–12 were performed at 10^6^ MCMC repetitions with a 10^5^ burn-in period using no prior information and assuming correlated allele frequencies and admixture. After the analysis was conducted, individual admixture proportions were sorted and displayed using DISTRUCT (Rosenberg, 2004). To determine the number of genetic clusters (K), we used the dealt K method (Evanno, Regnaut & Goudet, 2005) based on the second order rate of change in Ln Pr (X|K) as implemented in the program Structure Harvester version 0.6.94 (Earl & vonHoldt, 2012). Based on mtDNA CR sequence and nuclear DNA microsatellite data, migration rate (M) and effective population size (θ) were estimated using MIGRATE-n version 3.6.11 (Beerli, 2006; Beerli, 2007). In the MIGRATE analysis, we used Bayesian Inference or Maximum Likelihood and following parameters: slice sampling; uniform priors for θ between 0 and 0.2 and for M between 0 and 10,000; swapping among chains potentially occurring at every step; and 10 replicates. For each replicate, a burn-in of 10^4^ steps was followed by 10^7^, 3 × 10^7^ or 5 × 10^7^ parameter samplings recorded at intervals of 10^3^. All reported runs were met the convergence of criteria that an expected sample size (ESS) > 10^3^ and a good agreement of mean and median estimates for all parameters.

In the present study, demographic history based on microsatellites was assessed using the following methods. First, the Wilcoxon’s sign rank test was used to test for heterozygosity excess under the two-phase mutation model (TPM) and stepwise mutation model (SMM) (Cornuet & Luikart, 1996), with 95% single step mutations and 5% multi-step mutations. Second, a mode-shift test was carried out to detect any distortion of the expected L-shaped distribution of allele frequency (Luikart & Cornuet, 1998). Both Wilcoxon’s sign rank test and modeshift test were performed in BOTTLENECK 1.2.02 (Piry, Luikart & Cornuet, 1999). Finally, Markov-Switching Vector Autoregressions (MSVAR) (version 1.3), a MCMC simulation program, was used to provide estimates of the current and ancestral population size and the time since the population change (Beaumont, 1999; Storz, Beaumont & Alberts, 2002). The most important parameters for MSVAR are *N*_0_, *N*_1_, and *T*, where (1) *N*_0_ is the current effective population size, (2) *N*_1_ is the historical or ancestral effective population size, and (3) *T* is the time since the population change. Five independent simulations were ran to estimates the distributions of the three parameters (*N*_0_, *N*_1_ and *T*). The reported values of the tufted deer’s generation time are 1.5–2.5 years (Sheng et al., 1992), so here we set it as 2 years for the simulation. In every simulation, we ran each chain with a thinning interval of 10,000 steps, leading to a total number of Monte Carlo searches of 1 × 10^9^ steps with the first 10% of total iterations discarded as burn-in. The remaining data were used to obtain the lower (5%), the median (50%), and the upper (95%) quantiles of the posterior distributions. Different means for the average *N*_1_ were used to represent three demographic histories: (i) *N*_0_ > *N*_1_, an expanding population; (ii) *N*_0_ = *N*_1_, a stable population; and (iii) *N*_0_ < *N*_1_, a decreasing population. We estimated the marginal posterior distributions of the model parameters using the LOCFIT package (Loader, 2007) implemented in R v2.11.1 (R Development Core Team, 2009). At last, we combined the data from five runs to obtain the lower (5%), the median (50%), and the upper (95%) of the posterior distributions. The DBS population was ignored for MSVAR analysis due to the small sample size.

## Results

### Genetic diversity

We analyzed 1,170 bp of complete mtDNA CR and flanking sequences from 52 tufted deer after alignment (Table 1), and 39 haplotypes (HAP1–HAP39) were defined based on the target fragment. In the tufted deer, the overall haplotype diversity was 0.98 and the nucleotide diversity was 0.0228, which indicated a relatively high genetic diversity level compared with nine other deer species (Table 1).

**Table 1:** Genetic diversity of *Elaphodus cephalophus* and other deer species based on mtDNA CR sequence.

Species	n	nh	h	π	Reference
*Elaphodus cephalophus*	52	39	0.98	0.0228	In this study
Wulingshan population	36	27	0.98	0.0226	In this study
Dabashan population	16	15	0.99	0.0228	In this study
*Cervus nippon*	21	18	0.98	0.014–0.022	Nagata et al. (1999)
*Cervus eldii*	48	15	0.89	0.022	Balakrishnan et al. (2003)
*Capreolus capreolus*	728	161	0.971	0.011	Randi et al. (2004)
*Ozotoceros bezoarticus*	54	45	0.99	0.011–0.025	González et al. (1998)
*Hydropotes inermis*	40	18	0.923	0.0138	Hu, Fang & Wan (2006)
*Moschus berezovskii*	109	27	0.934	0.0453	Peng et al. (2009)
*Moschus moschiferus*	22	18	0.97	0.019	Kholodova & Prikhodko (2006)
*Muntiacus reevesi*	45/56	24/10	0.952/0.734	0.0168/0.0077	Shi et al. (2010)
*Muntiacus crinifrons*					Wu & Fang (2005)
Captive population	18	3	0.569	0.0021	
Wild population	26	10	0.862	0.0056	

**Note:**

n, number of sample size; nh, number of haplotype; h, haplotype diversity; π, nucleotide diversity.

In the present study, the level of genetic diversity was also estimated by 13 nuclear microsatellite loci (Table 2). Micro-Checker did not indicate null alleles or genotyping errors such as large allele dropout or stuttering. Linkage disequilibrium was only statistically significant in only a very small proportion of tests (5.12%). The average *H*_O_ was 0.781 (range: 0.500–1.000), the mean *H*_E_ was 0.823 (0.663–0.940), and the *PIC* ranged from 0.472 to 0.907 (average 0.787) (Table 2). Among the 13 loci, allelic richness ranged from 3.863 to 14.000, with the overall allelic richness across loci being 9.604 (Table 2).

**Table 2:** Genetic diversity indices for each of the 13 microsatellite loci in *Elaphodus cephalophus*.

Locus	WLS					DBS					All				
	N_A_	*H*_O_	*H*_E_	AR	*PIC*	*N*_A_	*H*_O_	*H*_E_	AR	*PIC*	*N*_A_	*H*_O_	*H*_E_	AR	*PIC*
Mreg03	8	0.707	0.740	6.100	0.695	7	0.722	0.663	6.495	0.615	9	0.712	0.720	6.123	0.681
Mreg22	14	0.780	0.890	10.191	0.867	10	0.667	0.844	9.051	0.800	16	0.746	0.880	10.589	0.861
Mreg25	8	0.732	0.729	5.919	0.678	4	0.813	0.563	3.863	0.472	8	0.754	0.697	5.447	0.647
Mreg26	9	0.707	0.734	6.032	0.680	8	0.722	0.702	7.022	0.632	11	0.712	0.723	6.195	0.672
Mre35	18	0.951	0.905	11.790	0.885	16	0.944	0.940	14.286	0.907	19	0.949	0.915	12.472	0.900
Mre39	9	0.683	0.778	7.083	0.740	8	0.611	0.775	7.629	0.725	11	0.661	0.785	7.702	0.755
Mre49	22	0.722	0.849	12.655	0.826	14	0.571	0.788	14.000	0.749	25	0.680	0.834	12.728	0.816
Mre61	15	0.971	0.918	12.049	0.897	13	0.813	0.917	12.327	0.879	19	0.922	0.918	12.488	0.902
Mreg143	13	0.756	0.847	9.343	0.820	9	0.889	0.852	8.287	0.808	14	0.797	0.854	9.021	0.830
Mreg252	12	1.000	0.827	8.860	0.797	13	1.000	0.876	11.524	0.838	15	1.000	0.840	9.529	0.816
Mreg260	16	0.829	0.898	11.954	0.877	9	0.722	0.822	8.066	0.773	18	0.797	0.876	11.240	0.857
Mreg283	15	0.659	0.824	10.057	0.798	11	0.500	0.838	9.791	0.793	18	0.610	0.831	10.035	0.808
Mreg284	19	0.829	0.916	12.987	0.898	13	0.944	0.867	11.311	0.826	21	0.864	0.900	12.303	0.884
Mean	13.7	0.794	0.835	9.617	0.805	10.4	0.763	0.804	9.512	0.755	15.7	0.785	0.829	9.682	0.802

**Note:**

*N*_A_, number of alleles; *H*_O_, observed heterozygosity; *H*_E_, expected heterozygosity; AR, allelic richness; *PIC*, polymorphism information content; WLS, Wulingshan population; DBS, Dabashan population; All, All individuals.

### Phylogeography, population structure and gene flow

**Figure 2:**
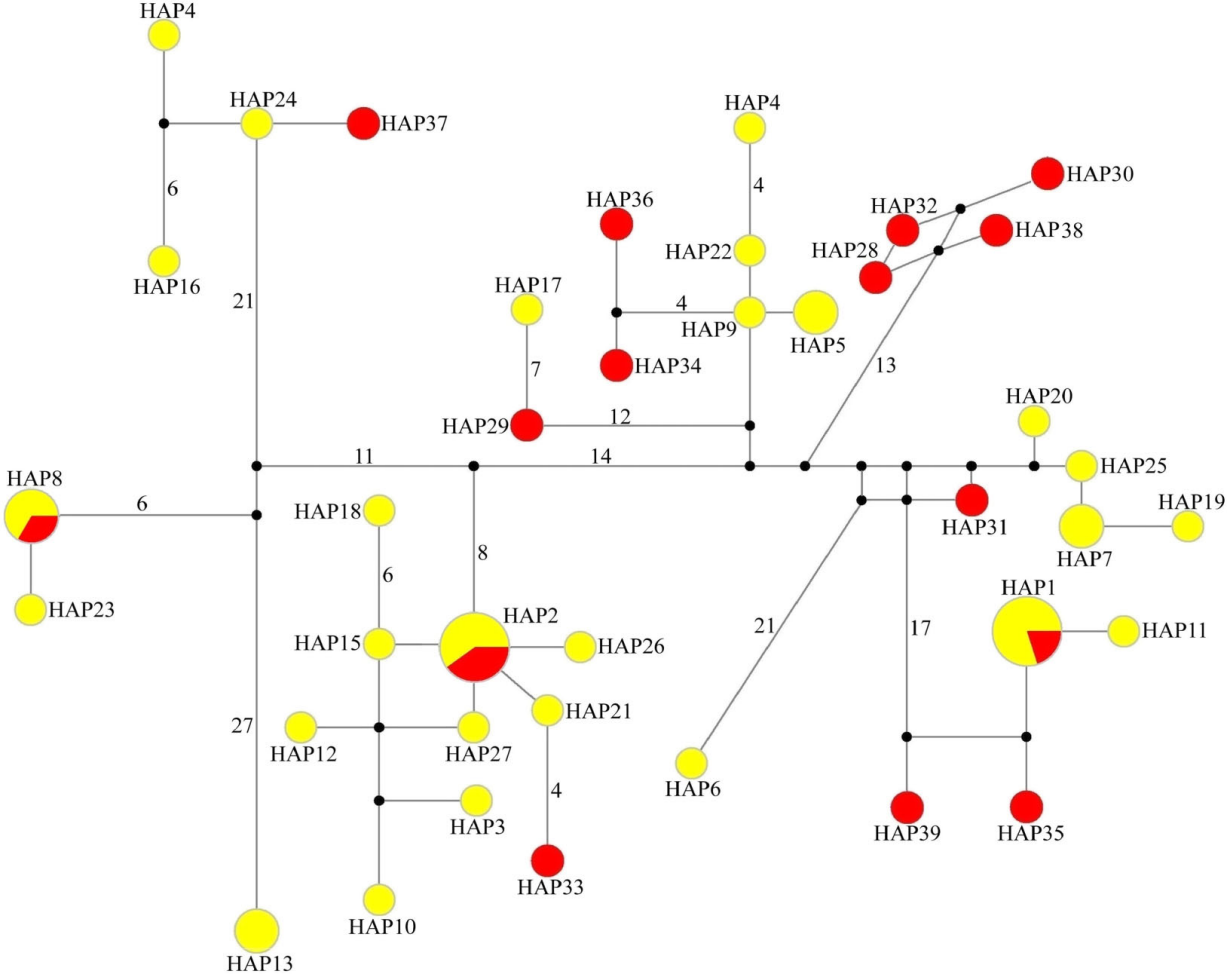
Median-joining network of 39 haplotypes found in tufted deer. In the network, the geographical origin of haplotypes is indicated by different colors (yellow-Wulingshan Mountain, red-Dabashan Mountain). Circles show the haplotype number and are proportional to the haplotype frequencies, black dots indicate undetected intermediate haplotype states. Connections with more than one nucleotide change are identified by numbers near the branches.

In the median-joining network of 39 haplotypes, three haplotypes (HAP1, HAP2 and HAP8) were shared by WLS and DBS, the other 36 haplotypes just belong to single population. Among them, WLS have 24 haplotypes and DBS have 12 haplotypes. In the network, the haplotypes from WLS mixed with that from DBS, and there are no distinct haplotype group corresponding to specific population (Fig. 2). Moreover, genetic differentiations (*F*_ST_) between WLS and DBS were minor, and without *statistical significance* (mtDNA CR: *F*_ST_ = 0.0115, *P* > 0.05; Microsatellite: *F*_ST_ = 0.0068, *P* > 0.05).

**Figure 3:**
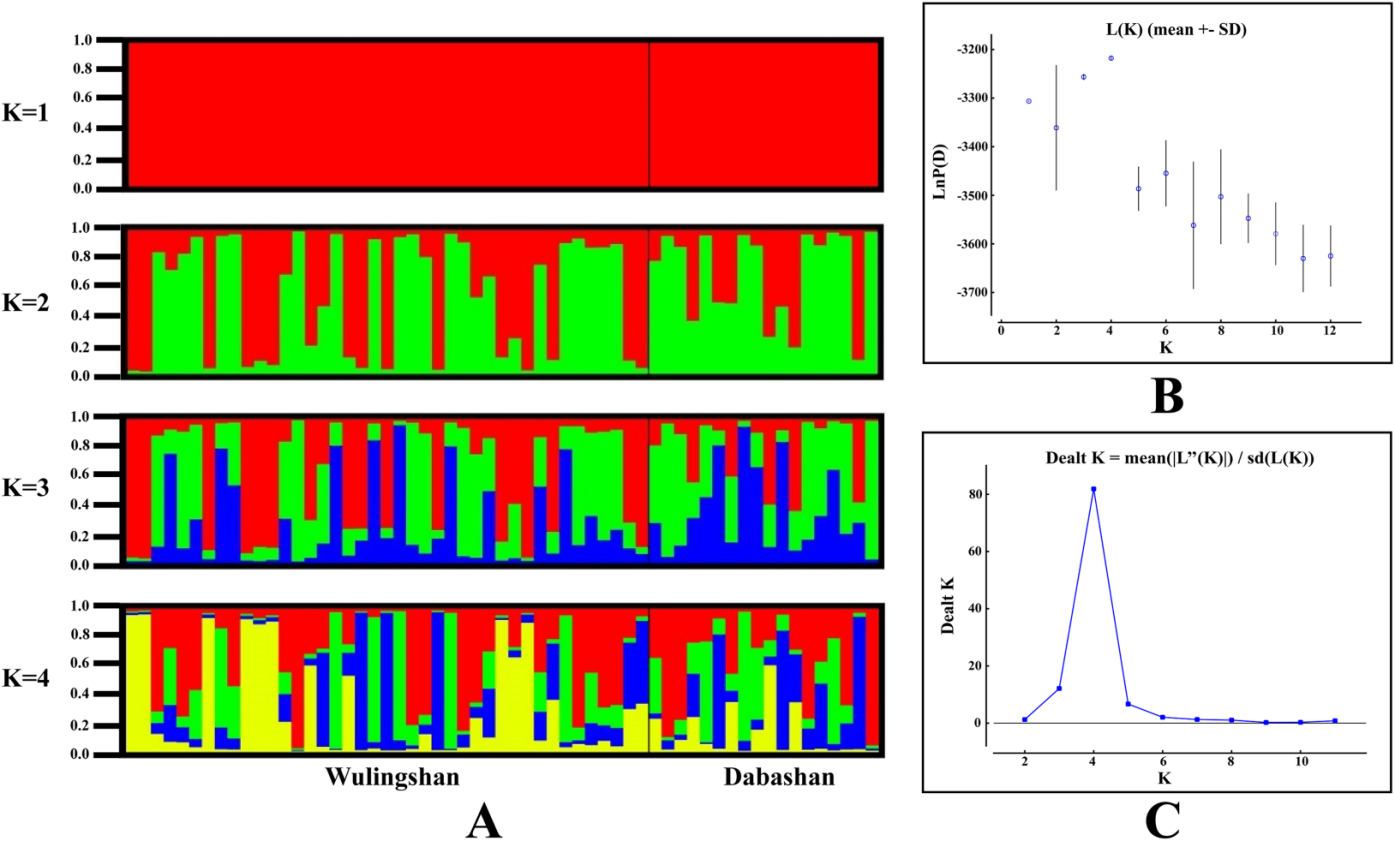
Bayesian cluster analysis of the microsatellite variation among two *Elaphodus cephalophus* populations. (A) Output of STRUCTURE analysis with population cluster (K) as 1, 2, 3 and 4. The proportion of ancestry assigned to each cluster was plotted by individuals. (B) the linear relationship between LnP(D) and K, (C) the rate of change of the likelihood function (Delta K).

**Figure 4:**
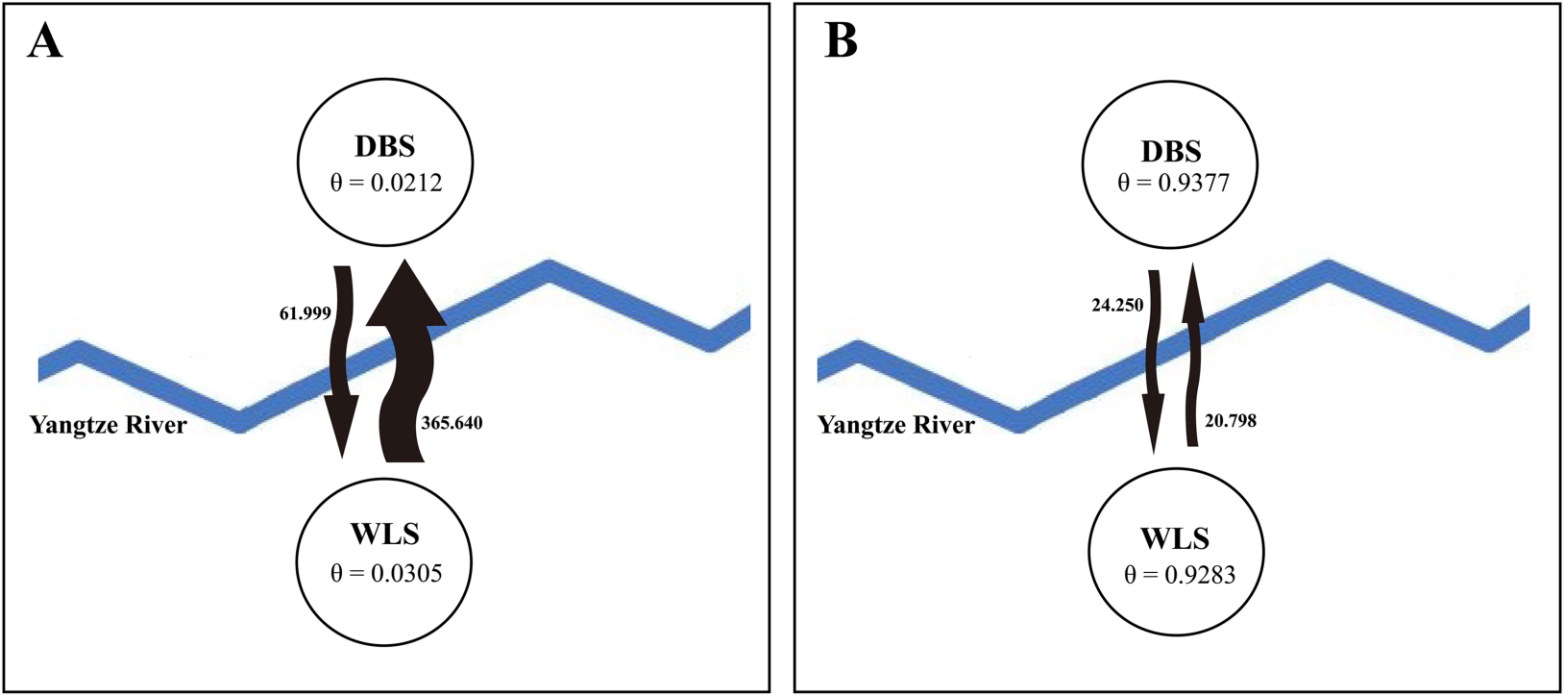
Likelihood estimates of gene flow and effective population size parameter (θ), based on (A) mtDNA CR sequence data and (B) microsatellite genotyping data. The arrows represent the direction of gene flow between populations, bold values beside the arrows represent maximum likelihood estimation (MLE) values of migration rate (M).

STRUCTURE analysis using multilocus microsatellite genotypes revealed a maximum posterior probability of the genetic data of K = 4 (Ln *P*(K) = −3,217.88) (Fig. 3). In addition, the Delta K value also showed a maximum (Delta K = 81.89) at K = 4 (Fig. 3). Therefore, it indicated that four potential genetic clusters exist in WLS and DBS populations. Noteworthily, some factors, such as recent admixture, admixture with unsampled/unobservable “ghost” populations, and recent bottleneck maybe lead to misinterpreting the STRUCTURE results (Gilbert et al., 2012; Lawson et al., 2012; Falush, Van Dorp & Lawson, 2016). On the other hand, although it seems that K = 4 is a reasonable result, it may at the same time be a psendophase. At the same time, there was no distinct genetic differences between the WLS or DBS population. The highly mixed color bars in the DISTRUCT diagram (for K = 2–4) indicated strong admixture between WLS and DBS populations. The gene flow test of the two populations verified the strong gene flow between them (Fig. 4). The analysis of mtDNA CR, showed strong and asymmetric gene flow between WLS and DBS. The value for maximum likelihood estimation (MLE) of migration rate from DBS to WLS is 61.999, and the value for MLE of migration rate from WLS to DBS is 365.640. However, in the analysis of microsatellite data, a symmetric gene flow pattern was found; the value for MLE of migration rate from DBS to WLS is 24.250, and the reversed value is 20.798. In the present study, when we conduct MIGRATE analysis with mtDNA CR and nuclear DNA microsatellite genotyping data, the values of θ is change correspondingly due to different data type, parameters and algorithm set. However, the nearly same effective population size (θ) were calculated in WLS and DBS, no matter using CR sequence data (θ = 0.0212 in DBS, 0.0305 in WLS) or microsatellite data (θ = 0.9377 in DBS, 0.9283 in WLS).

### Population demography

**Figure 5:**
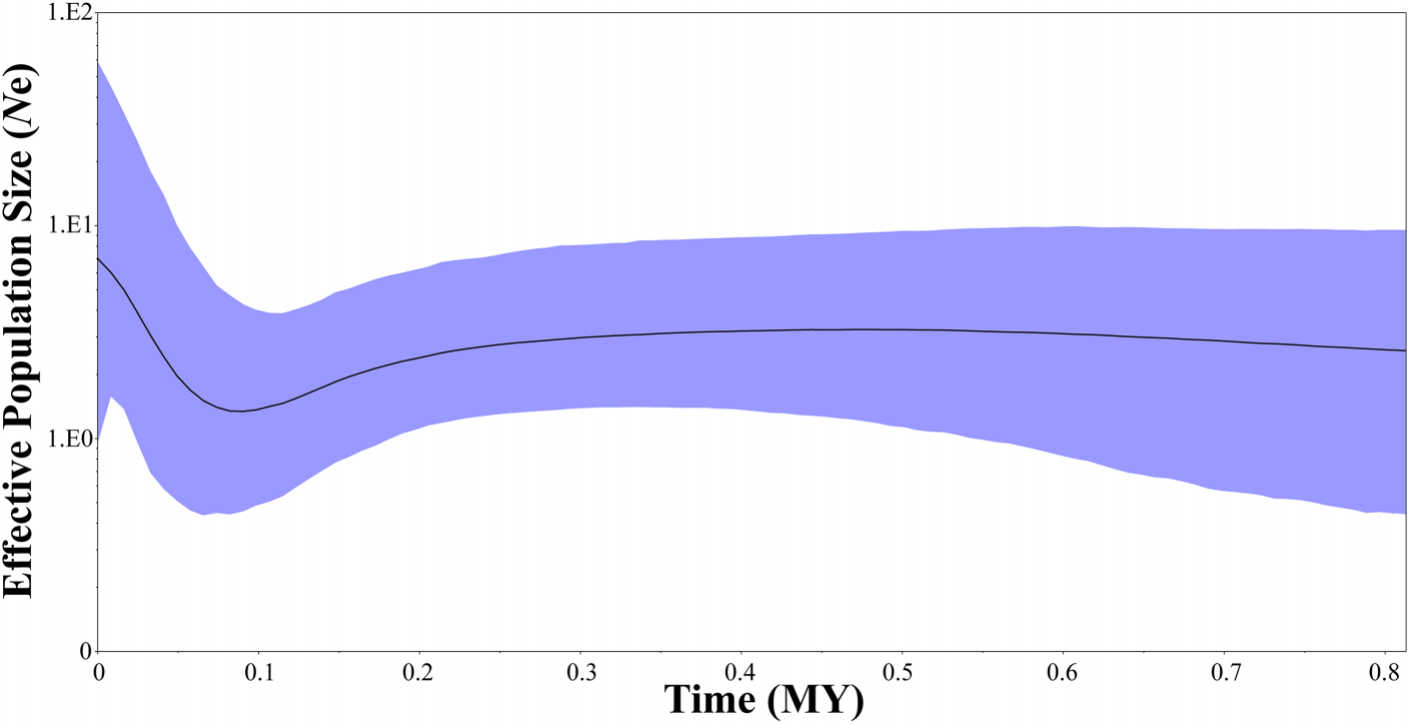
Bayesian skyline plots showing changes in female effective population size over time for *Elaphodus cephalophus*. Black curves represent median estimates of effective population size (Ne) through time, from the present to the time of the most recent common ancestor. The blue area represents 95% highest posterior density (HPD) limits.

Two neutrality tests were performed in WLS (Tajima’s *D* = 0.154, *P* = 0.612; Fu’s *Fs* = −1.506, *P* = 0.305) and DBS (Tajima’s *D* = −0.089, *P* = 0.506; Fu’s *Fs* = −1.688, *P* = 0.157), respectively. In the two neutrality tests, none of the calculated values were statistically significant, although most of the values were negative. However, BSP indicated a population fluctuation in the late Pleistocene. BSP suggested that tufted deer population size remained stable for quite a long time, from 0.8 to 0.3 million years before present, then it fell into a gradual decline from approximate 0.3–0.09 Ma BP, and experienced a expansion from approximately 0.09 Ma BP (Fig. 5).

**Figure 6:**
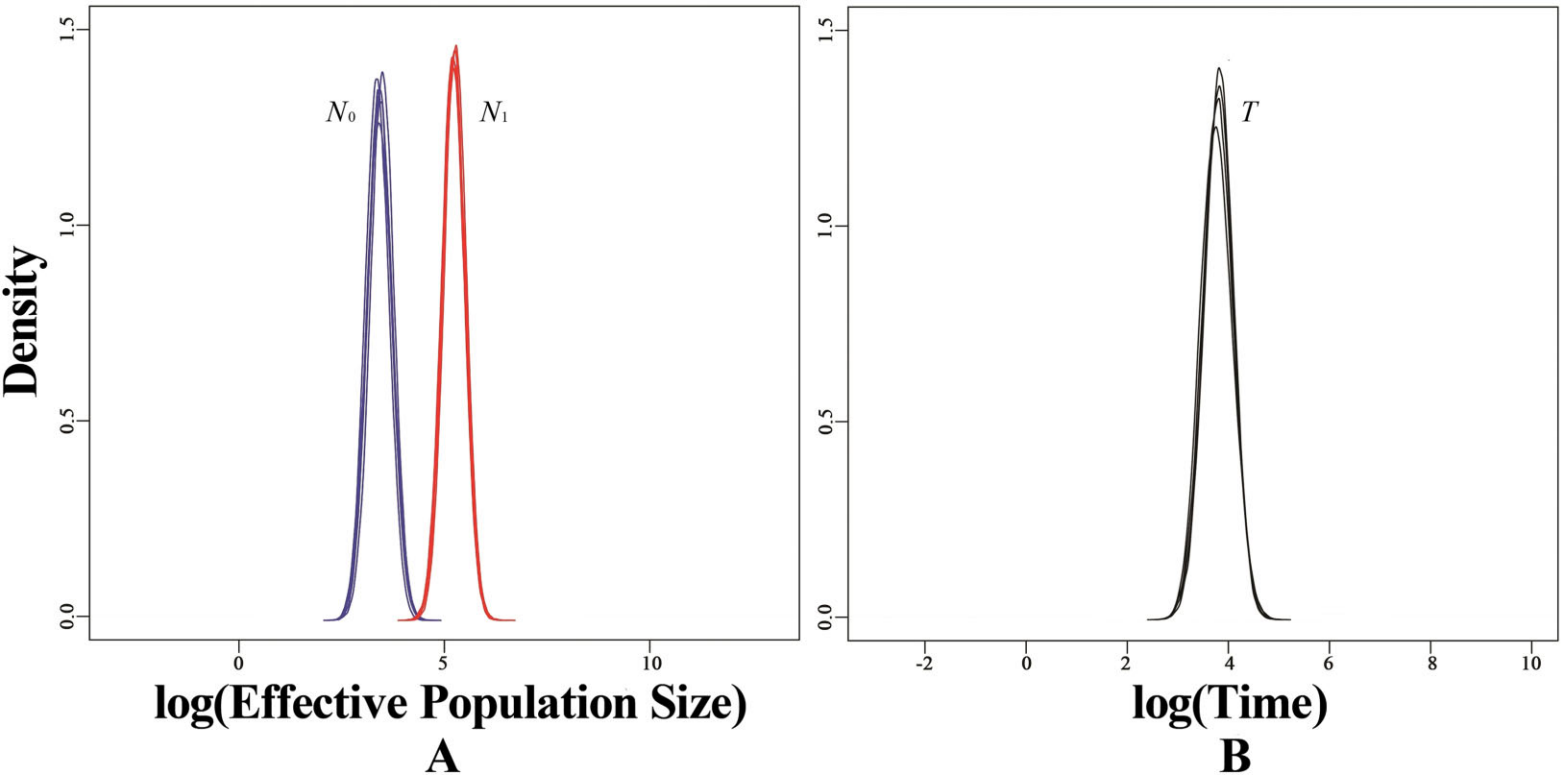
Estimated posterior distributions of *N*_0_, *N*_1_ and *T* using MSVAR. *N*_0_, current effective population sizes (blue curve); *N*_1_, ancestral effective population sizes (red curve); *T*, time since population change (black curve). All densities are represented in a log_10_ scale.

Based on microsatellite data, BOTTLENECK indicated no significant signal of recent bottleneck in WLS and DBS populations under both TPM and SMM. In addition, the mode-shift test showed a normal L-shaped distribution of allele frequencies. However, a strong signal of recent population decline was detected using MSVAR simulations in WLS population (Fig. 6A). MSVAR simulations also provided a consistent posterior distribution to the origin of population decline, with a median log *T* = 3.643 (Fig. 6B), so the most probable population decline time (*T*) in WLS population was 4,396 (5–95% quantiles: 1,428–15,393) years. The posterior distribution of *N*_0_ and *N*_1_ did not overlap in the exponential model, the median log *N*_0_ and log *N*_1_ values are 3.451 and 5.257 respectively (Fig. 6A); so the medians of the posterior distribution were approximately 2,824 (5–95% quantiles: 784–7,844) for *N*_0_ and 180,717 (5–95% quantiles: 56,885–458,277) for *N*_1_ (Fig. 6A). Therefore, it suggested a strong decline in WLS population (*N*_1_/*N*_0_ = 63.99).

## Discussion

### Genetic diversity

In this study, the haplotype and nucleotide diversities of the mtDNA CR in the tufted deer were higher than many of those other deer species (Table 1). The mean *H*_E_ (*H*_E_ = 0.823) and average *H*_O_ (*H*_O_ = 0.781) also showed high genetic diversity in 13 nuclear microsatellite loci (Table 2). These results indicated that these deer populations may still possess high genetic diversity, despite serious population decline. Species with great genetic diversity often had large population sizes and were geographically widespread in recent history (González et al., 1998; Balakrishnan et al., 2003). Therefore, in this study, the high genetic diversity of the tufted deer may indicate the large size of the ancestral populations. On the other hand, it also implied that the recent sharp population decline has not been severe enough to result in a significant loss of genetic diversity in the tufted deer, and the wild tufted deer populations may still harbor a surprisingly rich gene pool.

### The barrier effect of the Yangtze River

Up to now, few studies have attempted to evaluate the effect of the Yangtze River as a geographical barrier. In this study, the median-joining network of haplotypes did not show a clear pattern with a specific phylogeographic structure marked by the Yangtze River (Fig. 2). Furthermore, neither did STRUCTURE analysis identify genetic clusters corresponding to specific populations (Fig. 3). Conversely, the clustering results indicated unobstructed admixture between WLS and DBS population. Analyses in MIGRATE also revealed the strong gene flow between WLS and DBS, both in the maternally inherited mtDNA CR sequence, and the nuclear microsatellite loci genotype information (Fig. 4).

It has been reported that large, wide rivers can act as natural barrier to gene flow in various taxonomic groups, such as mammals (e.g. San Martin titi monkey *Callicebus oenanthe*, Ayres & Clutton-Brock, 1992; Bonobos *Pan paniscus*, Eriksson et al., 2004; Eastern chipmunk *Tamias striatus*, Chambers & Garant, 2010), reptiles (e.g. *Gymnodactylus darwinii* complex, Pellegrino et al., 2005) and even amphibians (e.g. Boulenger’s lazy toad *Scutiger boulengeri*, Li et al., 2009). Generally, rivers cannot limit gene flow in those species capable of swimming, such as the North American river otter *Lontra canadensis* (Blundell et al., 2002) and the American black bear *Ursus americanus* (Cushman et al., 2006). However, rivers were often considered as the important geographical barrier for deer, such as the white-tailed deer (*Odocoileus virginianus*, Robinson et al., 2012) and the red deer (*Cervus elaphus*, Pérez‐Espona et al., 2008). In the present study, the results indicated that the Yangtze River, at least in its middle reaches, did not act as an effective geographical barrier.

Previous studies showed that the Yangtze River exhibited flexible obstructing effect in different species. It is a more effective dispersal barrier for the house mice (*Mus musculus*) (Jing et al., 2014), but less significant for the wild boar (*Sus scrofa*) (Zhang et al., 2008). For the tufted deer, it is apparent that crossing a large river, such as the Yangtze River, is very difficult for its poor swimming ability (Sheng et al., 1992). So, the Yangtze River should be an effective barrier for the tufted deer. However, the barrier effect of rivers is unstable due to changing hydrological factors, for example, water level, width, flow rate, bed height and channel position (Wang et al., 2015). The Yangtze River, originates from the Tibetan Plateau and flows into the East China Sea, which contained the section partitioning Wulingshan mountains and Dabashan mountains (Chen et al., 2001). However, during the Last Glacial Maximum (LGM), the water level of the Yangtze River fell by about 20–45 m (Yang, 1986), which could have resulted in many crossing points in the up or middle reaches area. Similarly, historical arid climate events often led to the owering of the Yangtze River (Lu, 2014). So, potential water-level fluctuation made it possible that the tufted deer populations on the two sides crossed the Yangtze River freely. Therefore, the Yangtze River did not act as an effective genetic boundary between WLS and DBS populations. It seems that Yangtze River is not an insurmountable geographical barrier for some animals, at least in its middle reaches.

### Population demography

In the present study, we select two markers systems, mtDNA CR sequences and nuclear microsatellite markers to explore population demography in different time scales. In general, mtDNA CR sequences could provide the population demography information at longer time scale (e.g. from tens of thousands years to hundreds of thousands years) (Jing et al., 2014; Pan et al., 2014; Zhang et al., 2014; Wang et al., 2015). In contrast, nuclear microsatellite loci is usually good at detecting relatively recent population events (e.g. within several thousand years) (Zhang et al., 2007; Hu et al., 2011; Wang et al., 2015). Therefore, these two genetic markers systems provide different population demography information in different time scales (Hu et al., 2011; Wang et al., 2015). In fact, what they reflected were different population events in the evolutionary history of tufted deer.

In this study, a historical population decline signal was detected at approximate 0.3–0.09 Ma BP (Fig. 5), followed by a rapid population expansion (from 0.09 Ma BP to now, Fig. 5). The population decline is consistent with Guxiang Glaciations, the penultimate glaciation in Tibetan Plateau and surrounding mountains, which began from 0.36 Ma BP and lasted to 0.125 Ma BP (Ou et al., 2015). The Guxiang Glaciations was followed by the Last Interglacial (0.125–0.075 Ma BP) and the Dali Glaciations (Last glacial period, 0.075–0.011 Ma BP). It is noteworthy that the population demography of tufted deer was inconsistent with these typical species in North America and Europe, such as the saltmarsh sparrow *Ammodramus caudacutus* (Avise & Walker, 1998), the brown bear *Ursos arctos* (Hewitt, 2000) and the Western European Hedgehog *Erinaceus europaeus* (Hewitt, 2004). In these species, the population expansion events often occurred after the LGM (0.022–0.018 Ma BP). In the Yangtze River basin, many native species had experienced strong expansion, such as the red knobby newt *Tylototriton shanjing* (Yu et al., 2013), the Yunnan spiny frog *Nanorana yunnanensis* (Zhang et al., 2010) and the grey-cheeked fulvetta *Alcippe morrisonia* (Song et al., 2009). However, these expansion events all happened much earlier than the LGM. As a typical herbivore, the tufted deer is very sensitive to natural environment changes. During the Guxiang Glaciations, the mean annual air temperature decline would have been close to 7.8 °C (Zhou et al., 2010; Shi, Zhao & Wang, 2011). Inevitably, the cold climatic conditions exerted huge influence on natural environment. Previous study suggests that insolation rise triggered the final great climate rises during the Last Interglacial (Yuan et al., 2004). Hence, we consider that the population decline in the tufted populations may be attributed to the cold weather in the Guxiang Glaciations, and the warmer weather in the Last Interglacial might have triggered subsequent rapid population expansion.

The wild populations of tufted deer have declined sharply because of overhunting and habitat degradation (Zhang & Wei, 2007). However, BOTTLENECK detected no evidence of recent population bottleneck using both heterozygosity excess test and mode-shift test. But MSVAR analysis disclosed a recent signal of severe population decline in the WLS population (Fig. 6). The posterior distributions of *N*_1_ (median *N*_1_ = 180,717) and *N*_0_ (median *N*_0_ = 2,824) indicated an approximate 64-fold effective population decline in the WLS population (Fig. 6). Moreover, simulations also indicated this population decline started several thousand years ago (*T* = 4,396 years). In last decades, it was confirmed that in many species, the recent population demography events had close relationship with mankind’s activities (Goossens et al., 2006; Zhang et al., 2007; Hu et al., 2011; Zhu et al., 2011; Wang et al., 2015). In Northeastern Borneo, human-induced deforestation and habitat fragmentation resulted in a recent demographic collapse in the orang-utans *Pongo pygmaeus* (Goossens et al., 2006). Similarly, strong population decline was found in the giant panda, which triggered by the expansion of human populations several thousand years ago (Zhang et al., 2007). In addition, in the red panda *Ailurus fulgens* and the Yunnan caecilian *Ichthyophis bannanicus*, a similar recent population decline was also been found (Hu et al., 2011; Wang et al., 2015), all cases representing how wildlife suffered demographically at the hands of ever-increasing human pressure. Therefore, the recent population decline in the tufted deer WLS population may have been triggered by the development of early human activities and civilization.

Fortunately, a relatively high genetic diversity is still present in the tufted deer populations, but further studies focusing on population conservation are desirable. In view of the current population decline trend, appropriate protection and management strategies should be considered to maintain the present tufted deer populations.

## Supplemental Information

10.7717/peerj.2654/supp-1 Supplemental Information 1 Raw data exported from the ABI PRISM 3730 genetic analyzer (Applied Biosystems) with a GS500 size standard, and analyzed using GENEMARKER.

## Additional Information and Declarations

### Data Deposition

The following information was supplied regarding data availability: The following information was supplied regarding the deposition of DNA sequences: GenBank (accession numbers KT152891–KT152929).
