# Supplementary material for: Yangtze River, an insignificant genetic boundary in tufted deer (Elaphodus cephalophus): the evidence from a first population genetics study
Source: PeerJ. 2016 Nov 8;4:e2654. doi: 10.7717/peerj.2654 (PMC5103815; doi:10.7717/peerj.2654)
Supplement: Supplemental Information 1 [file peerj-04-2654-s001.pdf]

	Mreg-03-T		Mreg-22-H		Mreg-25-F		Mreg-26-T	
WLS1	129	129	251	265	282	286	180	184
WLS2	129	129	251	265	282	282	180	184
WLS3	125	131	249	267	274	278	166	182
WLS4	131	133	265	265	274	278	166	166
WLS5	133	143	265	267	274	278	166	166
WLS6	131	133	265	267	274	278	166	178
WLS7	131	131	267	271	282	282	166	180
WLS8	131	143	249	287	274	278	166	180
WLS9	131	141	249	287	278	280	166	182
WLS10	125	131	249	271	278	278	184	184
WLS11	131	133	267	271	282	282	166	180
WLS12	131	131	249	267	278	282	166	180
WLS13	131	133	249	251	282	282	180	184
WLS14	143	149	249	265	274	278	168	200
WLS15	129	131	281	287	274	278	180	184
WLS16	133	135	249	251	274	278	180	180
WLS17	133	143	287	289	278	282	184	184
WLS18	133	143	249	267	278	282	166	180
WLS19	133	141	251	291	274	278	180	180
WLS20	131	131	267	287	278	288	166	166
WLS21	133	143	251	291	274	278	166	180
WLS22	133	143	249	265	284	284	166	180
WLS23	131	133	249	265	278	280	166	166
WLS24	131	143	267	267	278	282	166	174
WLS25	131	133	251	291	274	278	166	180
WLS26	133	143	249	287	278	282	184	184
WLS27	131	133	265	285	278	286	166	178
WLS28	131	133	267	267	282	282	166	180
WLS29	131	131	249	251	274	278	166	198
WLS30	131	131	259	259	282	282	166	174
WLS31	141	143	267	267	286	294	180	180
WLS32	131	131	271	281	282	282	184	184
WLS33	141	143	251	269	278	284	166	180
WLS34	141	141	281	281	274	278	180	182
WLS35	131	133	259	259	278	278	166	180
WLS36	131	143	249	265	278	278	166	180
WLS37	129	133	267	287	278	282	166	174
WLS38	131	131	249	255	274	278	166	184
WLS39	131	133	283	285	274	278	180	180
WLS40	131	131	281	281	278	288	166	180
WLS41	131	131	281	281	274	278	180	184
DBS1	129	131	267	267	278	278	166	194
DBS2	131	133	249	265	274	278	166	180
DBS3	131	143	265	265	274	278	180	184
DBS4	131	131	249	269	274	278	166	184
DBS5	129	143	267	267	274	278	166	166
DBS6	131	141	249	265	278	278	180	180
DBS7	131	143	249	287	274	278	166	180
DBS8	133	143	265	265	278	284	166	180
DBS9	131	131	249	271	278	278	166	180
DBS10	131	135	249	267	278	282	166	180
DBS11	131	141	249	269	278	284	180	180
DBS12	131	131	267	267	274	278	174	180

DBS13	129	131	255	255	274	278	166	182
DBS14	131	131	249	255	?	?	166	182
DBS15	131	143	283	287	274	278	166	166
DBS16	131	131	249	303	274	278	166	166
DBS17	131	133	249	269	?	?	166	180
DBS18	143	147	249	263	274	278	200	224

Mre-35-T		Mre-39-H		Mre-49-F		Mre-61-F		Mreg-143-F
192	202	241	241	248	276	297	299	194
192	202	241	241	276	276	297	299	194
182	202	217	225	276	276	295	297	196
182	204	217	227	256	276	283	297	200
192	204	233	233	238	240	285	291	198
204	212	225	243	256	276	?	?	196
184	190	225	241	256	256	291	297	182
176	200	219	219	256	276	305	307	196
182	212	217	217	?	?	305	307	186
200	202	227	243	256	276	297	313	198
200	206	225	241	238	240	299	307	194
190	190	225	241	256	276	291	295	182
190	204	225	233	?	?	313	321	198
178	188	249	253	?	?	?	?	168
192	202	225	241	336	338	261	261	204
200	218	219	243	346	348	273	275	194
188	192	217	217	250	276	305	307	194
192	200	217	225	276	278	313	315	198
190	214	217	225	276	276	293	295	196
192	204	217	227	250	276	297	307	196
202	214	217	227	344	346	?	?	198
176	194	217	227	256	276	305	307	194
190	202	217	227	254	276	313	315	190
192	200	217	233	272	274	283	285	182
202	214	217	227	344	346	?	?	198
188	192	217	217	250	250	305	307	194
200	204	217	243	358	360	293	295	198
202	204	217	217	336	338	283	315	184
190	204	217	225	?	?	297	307	198
190	210	225	225	278	278	297	299	198
190	208	225	243	256	258	297	313	184
192	214	217	225	256	256	295	297	198
190	204	217	225	276	276	261	291	198
200	202	241	243	276	276	261	307	200
188	192	217	217	250	276	?	?	198
190	196	217	225	250	276	283	291	200
190	204	217	217	334	354	?	?	196
200	206	217	217	?	?	285	313	196
190	194	217	225	364	366	291	307	196
190	206	217	225	346	348	283	291	198
192	192	225	225	276	276	293	297	196
176	188	217	245	?	?	313	315	194
186	194	225	239	262	262	293	295	194
182	202	217	217	248	276	289	319	196
192	192	217	233	256	276	297	313	198
192	208	217	217	274	276	299	315	190
184	204	227	227	?	?	281	283	196
190	206	227	243	362	364	297	299	194
176	194	217	227	276	276	307	307	194
184	190	217	217	250	276	305	319	182
192	206	217	225	258	258	295	307	198
184	204	227	227	?	?	283	283	196
190	200	225	227	276	278	283	307	194

200	204	217	233	276	276	283	283	196
188	218	219	219	276	276	295	297	196
188	212	217	217	342	344	?	?	196
188	190	217	239	?	?	297	303	186
190	192	217	227	346	348	?	?	196
194	196	219	245	276	276	295	307	182

	Mreg-252-H		Mreg-260-F		Mreg-283-T		Mreg-284-F	
202	195	197	331	347	190	204	210	214
202	201	203	331	347	190	204	210	214
200	195	207	349	349	212	228	164	212
200	195	197	331	339	156	164	206	206
200	195	233	339	339	156	156	214	214
208	195	217	329	363	156	164	206	256
200	195	201	317	331	156	218	206	258
202	195	205	329	331	156	164	184	226
200	197	199	339	341	156	204	202	214
198	195	197	315	349	190	190	210	258
198	189	201	333	347	200	200	208	208
200	195	201	331	349	156	216	206	258
198	195	207	331	341	212	228	212	212
168	161	209	305	305	170	234	234	234
212	189	197	331	347	198	204	200	210
198	195	201	329	329	156	228	196	214
196	201	217	331	363	228	228	202	212
202	189	195	337	349	228	232	210	214
202	189	195	333	337	156	156	196	214
198	195	203	339	341	164	164	200	200
202	189	195	329	333	156	156	206	214
198	195	201	329	329	164	164	184	218
200	161	195	331	343	188	228	200	222
198	189	197	339	341	228	232	196	214
202	195	201	329	333	156	156	206	214
196	161	209	331	365	156	228	202	212
208	195	201	329	349	156	156	214	226
198	195	197	343	345	156	232	196	206
198	195	197	331	351	228	228	212	212
200	197	203	329	329	156	200	198	258
194	195	197	329	331	156	204	198	206
198	197	201	315	329	222	232	206	214
198	197	203	331	339	164	198	202	216
204	161	195	329	337	156	156	204	206
204	195	201	341	363	156	228	202	212
212	161	209	315	331	188	198	198	214
196	189	195	333	341	156	156	184	218
202	0	0	331	365	170	228	198	206
202	195	197	315	331	156	164	196	200
198	189	195	317	351	156	204	184	210
196	197	199	329	329	156	156	206	214
196	195	197	329	329	190	204	184	206
194	197	199	331	343	156	156	194	226
200	189	217	339	341	222	232	210	214
202	189	195	329	329	170	228	200	214
196	161	195	331	339	164	164	200	202
202	195	201	331	341	204	204	206	212
200	161	197	329	331	156	228	206	210
198	193	195	329	329	164	164	184	218
200	195	207	331	331	164	164	214	214
200	195	197	329	339	164	190	204	206
202	195	197	331	341	156	204	206	212
194	225	227	329	333	156	156	206	214

202	203	207	327	331	170	268	212	240
198	189	203	329	341	156	156	196	212
198	161	197	325	325	164	164	206	212
196	189	195	329	341	156	182	206	212
202	195	197	337	339	156	156	206	214
192	199	205	331	339	170	180	206	214
